# Zero-Temperature, Mean-Field Theory of Atomic Bose-Einstein Condensates

**DOI:** 10.6028/jres.101.055

**Published:** 1996

**Authors:** Mark Edwards, R. J. Dodd, Charles W. Clark, K. Burnett

**Affiliations:** Department of Physics, Georgia Southern University, Statesboro, GA 30460-8031; National Institute of Standards and Technology, Gaithersburg, MD 20899-0001; National Institute of Standards and Technology, Gaithersburg, MD 20899-0001; Clarendon Laboratory, Department of Physics, University of Oxford, Parks Road, Oxford OX1 3PU, United Kingdom

**Keywords:** Bogoliubov equations, Bose-Einstein condensation, Gross-Pitaevskii equation, linear excitations, mean-field theory, nonlinear Schrödinger equation, superfluidity, vortex formation

## Abstract

We review the application of zero-temperature, mean-field theory to current experimental atomic Bose-Einstein condensates. We assess the validity of the approximations made by comparing the mean-field results with a variety of experimental data.

## 1. Introduction

The recent reports of Bose-Einstein condensation (BEC) in weakly interacting trapped alkali gases [1–3] has confirmed a property of bosons first predicted in 1924 by Bose [4] for photons, and in 1925 by Einstein [5] for atoms. The production of such condensates has opened the possibility of a new generation of atomic physics experiments on meso- or macroscopic assemblies of atoms in the same quantum state.

The pure BEC phenomenon is manifested in the quantum statistical mechanics of noninteracting Bose particles; when encountered in nature it is modified by the effects of particle interaction. Such effects are quite severe in the case of superfluid ^4^He, which heretofore has been thought of as the canonical example of BEC: the strong interactions between ^4^He atoms allow only about 10 % of the atoms to occupy the condensate state [6]. However, it has recently been shown [7] that on the periphery of ^4^He droplets, the condensate fraction approaches unity; this is because the atomic density vanishes at the extremity of the droplet, so the atom-atom interaction energy also tends to zero. The recent alkali BECs are distinguished from liquid ^4^He in that the alkali atomic density is subject to wide-ranging experimental control, and has been indeed taken into the regime in which a dilute gas approximation is applicable, and in which it is reasonable to think of all atoms as occupying the same quantum state, as in the original concept of BEC. However, as will be seen below, the condensate state is much different from that which would describe a noninteracting gas. It is modified by effects of interactions that are encapsulated in a mean-field description, which can be thought of as the Hartree approximation to the wavefunction of a system of Bose particles. The quantitative treatment of such systems is the subject of this paper.

Before proceeding, we wish to note three additional aspects of the alkali systems that contrast with the case of liquid ^4^He, as these make the general approach to the problem somewhat different from those traditionally used to treat superfluid systems.

First, the alkalis are confined by an external potential (a magnetic field or combination of magnetic and light fields), so their density is inhomogeneous. Thus the alkali BECs cannot be described adequately by a spatially uniform condensate wavefunction such as that which is used to describe bulk liquid ^4^He. Not only must quantitative modelling methods be modified to treat inhomogeneous vs homogeneous BECs, but there are qualitative differences as well: for negative scattering lengths, a small long-lived BEC can exist in the inhomogeneous case [8,9], but not in a homogeneous system [10].

Second, as discussed by Cornell [11], the alkali BECs are intrinsically *metastable*. The equilibrium state of a confined alkali system at sub-microKelvin temperatures is a solid. However, the time for recombination of the gas is very long in the dilute limit, and is at least of the order of seconds in the current experiments.

Third, as shown elsewhere in this Special Issue [12–14], the ultracold collision physics of alkali BECs is exceedingly complex. Although the effects of collisions can be encapsulated in a few parameters (scattering lengths), the quantitative determination of these parameters is quite difficult and remains an active area of research. The work described in this paper utilizes these parameters as basic input, and it should be kept in mind that their values are subject to significant uncertainties, in no case less than 10 %.

This paper presents a partial review of work we have undertaken to date in the field of modeling the alkali BECs. This work has been based on a zero-temperature, mean-field formulation of the quantum mechanics of an externally confined system of weakly interacting Bose particles. Many of the results of this theory, such as condensate geometries, lifetimes, and excitation frequencies, can be directly compared with the data of current experiments. We shall use this comparison to assess the validity of applying mean-field theory (MFT) to the current crop of experimental Bose-Einstein condensates (BECs).

The zero-temperature MFT equations presented here were first derived by Bogoliubov [15] many years ago in order to study the superfluid ^4^He. The system to which they apply is assumed to be a weakly interacting, dilute gas of identical bosons, which, as noted above, does not provide a good description of liquid helium. However, it *seems* to fit the conditions present in a system of magnetically trapped gas of neutral alkali atoms. We emphasize that the previous statement should not be taken to be true *a priori*, but rather must be subjected to stringent experimental tests. We present here an overview of the comparison of MFT predictions with experiment.

The plan of the paper is as follows. In Sec. 2 we present a derivation of the Gross-Piteavskii (GP) and Bogoliubov equations (which we have been calling here the “MFT” equations). As part of the discussion we shall attempt to provide a detailed description of all of the approximations made in arriving at the MFT equations. In Sec. 3 we present the results of solving these equations for cases where comparison with experiment is possible. Sections 4 and 5 describe the algorithms and numerical procedures we have used to obtain the results presented in this paper and in previous work cited therein. Actual solution of the MFT equations for cases of specific experimental interest is a subject that has developed quite recently, and we believe there is considerable scope for enhancement of computational efficiency over that attained in current practice. Such enhancements will certainly be needed to go beyond the zero-temperature MFT description of BEC. Thus the material in Secs. 4 and 5 is presented at a level of detail needed to document our approach for use by those who can improve upon it.

## 2. Mean-Field Theory: Approximations and Derivations

In this section we present a somewhat detailed derivation of the basic zero-temperature MFT equations. These equations consist of the Gross-Pitaevskii equation, which describes the properties of the condensed part of the trapped atomic cloud, and the Bogoliubov equations, which describe properties of the non-condensed part. We shall present two derivations of the MFT equations. The first derivation uses a Bogoliubov transformation to cast the grand-canonical hamiltonian for a collection of interacting bosons into the form of a collection of noninteracting quasi-particles with the condensate becoming the vacuum state. The second derivation uses linear-response theory [16] performed on the *time-dependent* Gross-Pitaevskii equation (which is itself derived from a variational principle) to obtain the basic MFT equations. Before presenting these derivations, we shall first discuss the fundamental approximations made in modeling a cloud of cold, trapped atoms.

### 2.1 Fundamental Approximations

In the current generation of BEC experiments [1–3], a cloud of alkali atoms is optically pre-cooled and then magnetically trapped and evaporatively cooled to very low temperatures. The first major approximation leading to the MFT description is that the internal states of the atoms are ignored. All of the atoms must, however, reside in a particular hyperfine atomic ground state in order to remain trapped. The direction of the magnetic moment associated with the atom’s internal state has been polarized to lie along the direction of the trap magnetic field at the site of the atom. Since the atoms are very cold and thus slowly moving, we assume that the magnetic moment of the atom adiabatically follows the local magnetic field [17]. Thus the energy of the interaction of the atom’s magnetic moment (*μ*_atom_) with the external magnetic field has the form

$$V_{\mathrm{trap}}(r)=\mu_{\mathrm{atom}}B(r).$$ (1)

Another feature of this assumption is that collisions between atoms in the cloud do not change the atom’s internal state. That is, all collisions are assumed to be *elastic*. In fact, most inelastic (spin-flip) binary collisions will cause both atoms to be ejected from the trap. This, in turn, limits the lifetime of the condensate. Such lifetimes can be predicted within MFT in a reasonably accurate way for comparison with experiment. Such comparisons are presented below.

The true interaction potential between atoms in the cloud is quite complex. See, in this regard, Refs. [12–14] in this Special Issue. Most of this complexity is evident, however, only when the atoms are in close proximity. At the low temperature and density conditions present in the trap, all scattering events occur at extremely low energy. Consequently, the atoms rarely come close enough to each other to sample the complex nature of the inter-atomic potential. The atom-atom interaction is therefore well characterized by the *s*-wave scattering length, and the interaction potential may be written in the form:

$$V_{\mathrm{int}}(r-r^{\prime })=U_{0}\delta (r-r^{\prime })$$ (2)

where *U*_0_ = 4π*ħ*^2^*a*/*M*, *a* is the *s*-wave triplet scattering length, and *M* is the atomic mass.

In the next section we present the derivation of the MFT equations using the Bogoliubov prescription which begins with the assumption that the atomic cloud can be approximated by a restricted grand-canonical ensemble.

### 2.2 Bogoliubov Prescription

Consider the many-atom system whose temperature is well below the condensation point and which is composed of a condensate plus thermal atoms. The grand canonical, many-atom hamiltonian, $\hat{K}=\hat{H}-\mu \hat{N}$ where $\hat{H}$ is the many-body hamiltonian and $\hat{N}$ is the number operator, is written in terms of the field operator as follows:

$$\hat{K}=\int dr{\hat{\psi }}^{†}(r)H_{0}\hat{\psi }(r)+\frac{1}{2}U_{0}\int dr{\hat{\psi }}^{†}(r){\hat{\psi }}^{†}(r)\hat{\psi }(r)\hat{\psi }(r)-\mu \int dr{\hat{\psi }}^{†}(r)\hat{\psi }(r)$$ (3)

where *H*_0_ is the bare-trap hamiltonian,

$$H_{0}=-\frac{\hbar^{2}}{2M}\nabla^{2}+V_{\mathrm{trap}}(r),$$ (4)

*μ* is the chemical potential, and *V*_trap_(***r***) is the trap potential.

The boson field operators *ψ*^†^(***r***) and *ψ*(***r***), respectively create and destroy an atom at position ***r*** and satisfy the commutation relations.

$$\begin{array}{l} {}[\hat{\psi }(r),{\hat{\psi }}^{†}(r^{\prime })]=\delta (r-r^{\prime }),\\ {}[\hat{\psi }(r),\hat{\psi }(r^{\prime })]=[{\hat{\psi }}^{†}(r),{\hat{\psi }}^{†}(r^{\prime })]=0. \end{array}$$ (5)

Under the Bogoliubov approximation, the condensate is assumed to contain most of the atoms so that *N* − *N*_0_ << *N*_0_, where *N*_0_ denotes the macroscopic occupation of the condensate and *N* denotes the total number of condensate plus thermal atoms. In this case, the field operator can be written as the sum of a *c*-number condensate wave function, *Ψ*(***r***), plus a small correction, *ϕ*(***r***),

$$\hat{\psi }(r)=\Psi (r)+\hat{\phi }(r),$$ (6)

where *Ψ*(***r***) satisfies the normalization condition

$$\int dr|\Psi (r){|}^{2}=N_{0}.$$ (7)

Inserting Eq. (6) into Eq. (3) and neglecting terms in *ϕ*(***r***) higher than quadratic yields the following expression for $\hat{K}$.

$$\begin{array}{l} \hat{K}=\\ \int dr\Psi^{*}(r)[H_{0}-\mu +\frac{1}{2}U_{0}{\left|\Psi (r)\right|}^{2}]\Psi (r)\\ +\int dr\Psi^{*}(r)[H_{0}-\mu +U_{0}{\left|\Psi (r)\right|}^{2}]\hat{\phi }(r)\\ +\int dr{\hat{\phi }}^{†}(r)[H_{0}-\mu +U_{0}{\left|\Psi (r)\right|}^{2}]\hat{\psi }(r)\\ +\int dr{\hat{\phi }}^{†}(r)[H_{0}-\mu +2U_{0}{\left|\Psi (r)\right|}^{2}]\hat{\phi }(r)\\ +\frac{1}{2}U_{0}\int dr{\hat{\phi }}^{†}(r){\left(\Psi (r)\right)}^{2}{\hat{\phi }}^{†}(r)\\ +\frac{1}{2}U_{0}\int dr\hat{\phi }(r){\left(\Psi^{*}(r)\right)}^{2}\hat{\phi }(r). \end{array}$$

The first term in the above equation is a *c*-number and the second and third terms will vanish identically if *Ψ*(***r***) satisfies the GP equation [18]

$$[H_{0}+U_{0}|\Psi (r){|}^{2}]\Psi (r)=\mu \Psi (r).$$ (8)

The Bogoliubov-approximate grand canonical hamiltonian [15], ${\hat{K}}_{B}$, then takes the form

$$\begin{array}{l} {\hat{K}}_{B}=\zeta \prime +\int dr{\hat{\phi }}^{†}(r)[H_{0}-\mu +2U_{0}{\left|\Psi (r)\right|}^{2}]\hat{\phi }(r)\\ +\frac{1}{2}U_{0}\int dr{\hat{\phi }}^{†}(r){\left(\Psi (r)\right)}^{2}{\hat{\phi }}^{†}(r)\\ +\frac{1}{2}U_{0}\int dr\hat{\phi }(r){\left(\Psi^{*}(r)\right)}^{2}\hat{\phi }(r) \end{array}$$ (9)

where ξ is a *c*-number.

The Bogoliubov hamiltonian is a sum of a quadratic form and a *c*-number and can be cast into the form of a collection of noninteracting quasi-particles by the following Bogoliubov transformation [19]

$$\hat{\phi }(r)=\sum_{\lambda }\left(u_{\lambda }(r)\beta_{\lambda }+\upsilon_{\lambda }^{*}(r)\beta_{\lambda }^{†}\right)$$ (10)

and

$${\hat{\phi }}^{†}(r)=\sum_{\lambda }\left(u_{\lambda }^{*}(r)\beta_{\lambda }^{†}+\upsilon_{\lambda }(r)\beta_{\lambda }\right)$$ (11)

where the *β*_λ_ are quasi-particle creation and destruction operators and the implicit assumption is made that the condensate wave function is not included in the sum. The quasi-particle operators satisfy the usual commutation relations for boson creation and destruction operators

$$[\beta_{\lambda },\beta_{\lambda^{\prime }}^{†}]=\delta_{\lambda \lambda^{\prime }},\;[\beta_{\lambda },\beta_{\lambda^{\prime }}]=[\beta_{\lambda }^{†},\beta_{\lambda^{\prime }}^{†}]=0.$$ (12)

The reduction of ${\hat{K}}_{B}$ to a collection of noninteracting quasi-particles occurs if the *u*_λ_ and *ν*_λ_ satisfy the following equations (after setting $\Psi (r)=N_{0}^{1/2}\Psi_{g}(r)$)

$$ℒu_{\lambda }(r)+N_{0}U_{0}{\left(\psi_{g}(r)\right)}^{2}\upsilon_{\lambda }(r)=E_{\lambda }u_{\lambda }(r)$$ (13)

and

$$ℒ\upsilon_{\lambda }(r)+N_{0}U_{0}{\left(\psi_{g}^{*}(r)\right)}^{2}u_{\lambda }(r)=-E_{\lambda }\upsilon_{\lambda }(r)$$ (14)

where

$$ℒ=H_{0}-\mu +2N_{0}U_{0}{\left|\psi_{g}(r)\right|}^{2},$$ (15)

and the *u*_λ_ and *υ*_λ_ are square-integrable functions. The final form (to within a *c*-number) of ${\hat{K}}_{B}$ is

$${\hat{K}}_{B}=\sum_{\lambda }E_{\lambda }\beta_{\lambda }^{†}\beta_{\lambda }.$$ (16)

This hamiltonian has the form of a collection of noninteracting quasi-particles for which the condensate is the vacuum. Complete details of the derivation of the final form of ${\hat{K}}_{B}$ are given in Ref. [19]

### 2.3 Linear-Response Theory

The *time-dependent* Gross-Pitaevskii equation can be derived from an action principle if a “boson coherent state” is used as the trial wave function [20],

$$|\Phi (t)\rangle =\sum_{n=0}^{\infty }{\left(\frac{N_{0}^{n}}{n!}\right)}^{1/2}|\Phi_{n}(t)\rangle .$$ (17)

The factor |*Φ_n_*(*t*)〉 is the usual Hartree many-body trial wave function—an *n*-fold product of one single-particle orbital *Ψ*(***r***,*t*). The time-dependent GP equation describes the evolution of this orbital,

$$i\hbar \frac{\partial \Psi }{\partial t}=[H_{0}+U_{0}{\left|\Psi (r,t)\right|}^{2}]\Psi (r,t).$$ (18)

The basic MFT equations can be obtained by a standard linear-response analysis of this equation [16]. To this end, we consider the effect of adding a weak, sinusoidal perturbation to the trap potential. The time-dependent GP equation then has the form

$$i\hbar \frac{\partial \Psi }{\partial t}=[H_{0}+U_{0}{\left|\Psi (r,t)\right|}^{2}+f_{+}(r)e^{-i\omega_{p}t}+f_{-}(r)e^{i\omega_{p}t}]\Psi (r,t).$$ (19)

The *f*_±_(***r***) are the (possibly spatially dependent) amplitudes of the sinusoidal perturbation and *ω*_p_ is the probe frequency.

To find the linear response of the condensate to the driving field, we shall assume that *Ψ*(***r***,*t*) takes the form of a sum of an undisturbed ground-state part and a response part that oscillates at frequencies ±*ω*_p_:

$$\Psi (r,t)=e^{-i\mu t/\hbar }[N_{0}^{\frac{1}{2}}\psi_{g}(r)+u(r)e^{-i\omega_{p}t}+\upsilon^{*}(r)e^{i\omega_{p}t}]$$ (20)

Here, *μ* is interpreted as the chemical potential of the undisturbed ground state, and the condensate wavefunction is represented by the (scaled) condensate orbital *ψ_g_*(***r***). The functions *u*(***r***) and *υ*(***r***) are the components of the condensate’s linear response to the external disturbance that oscillate at frequencies ±*ω*_p_.

After inserting Eq. (20) into Eq. (19), retaining only terms up to first-order in *u*(***r***), *υ*(***r***), and *f*_±_ and equating like powers of $e^{\pm i\omega_{p}t}$, there result three equations that must be simultaneously solved for *ψ_g_*(***r***), *u*(***r***), *υ*(***r***), and *μ*. These equations describe the linear response of the condensate to weak external perturbation and have the following form

$$[H_{0}+N_{0}U_{0}{\left|\psi_{g}(r)\right|}^{2}]\psi_{g}(r)]=\mu \psi_{g}(r),$$ (21)

$$[ℒ-\hbar \omega_{p}]u(r)+N_{0}U_{0}{\left(\psi_{g}(r)\right)}^{2}\upsilon (r)=-N_{0}^{1/2}f_{+}(r)\psi_{g}(r),$$ (22)

$$[ℒ+\hbar \omega_{p}]\upsilon (r)+N_{0}U_{0}{\left(\psi_{g}^{*}(r)\right)}^{2}u(r)=-N_{0}^{1/2}f_{-}^{*}(r)\psi_{g}^{*}(r).$$ (23)

To make the final connection with the basic equations of MFT, we show that Eqs. (22) and (23) can be solved by writing their solution as an expansion in the condensate normal modes. The equations that determine these modes are identical to the Bogoliubov equations. We now discuss there solution.

To find the normal modes of the condensate, we first set *f*_±_(***r***) to zero in Eqs. (22) and (23). It is clear that the resulting equations will support square-integrable solutions only for discrete values of *ω*_p_, (we shall label them as *ω*_λ_). The normal-mode equations thus have the form

$$[ℒ-\hbar \omega_{\lambda }]u_{\lambda }(r)+N_{0}U_{0}{\left(\psi_{g}(r)\right)}^{2}\upsilon_{\lambda }(r)=0,$$ (24)

and

$$N_{0}U_{0}{\left(\psi_{g}^{*}\;(r)\right)}^{2}u_{\lambda }(r)+[ℒ+\hbar \omega_{\lambda }]\upsilon_{\lambda }(r)=0.$$ (25)

where λ represents a set of quantum numbers. These equations are identical to Eqs. (13) and (14) if *E*_λ_ = *ħω*_λ_. To complete the connection between the quasi-particle excitation spectrum and the condensate response we now show how these normal modes describe the condensate linear response.

We define a *normal mode* as the following two-component object:

$$\phi_{\lambda }(r)=\left(\begin{array}{c} u_{\lambda }(r)\\ \upsilon_{\lambda }(r) \end{array}\right).$$ (26)

With this definition, Eqs. (24) and (25) can be cast in the form,

$$H\phi_{\lambda }(r)=\hbar \omega_{\lambda }\sigma_{3}\phi_{\lambda }(r).$$ (27)

Where

$$H=\left(\begin{array}{cc} ℒ & V\\ V^{*} & ℒ \end{array}\right),\;\sigma_{3}=\left(\begin{array}{cc} 1 & 0\\ 0 & -1 \end{array}\right),$$ (28)

with *V*(***r***) = *N*_0_*U*_0_(*ψ_g_*)^2^(***r***) and ℒ is defined by Eq. (15).

The {*ϕ*_λ_} form a complete [20] orthonormal set where the scalar product of two normal modes is defined by

$$\langle {\phi_{\lambda }}_{1}|{\phi_{\lambda }}_{2}\rangle \equiv \int dr\phi_{\lambda_{1}}^{†}(r)\sigma_{3}{\phi_{\lambda }}_{2}(r)=\delta_{\lambda_{1}\lambda_{2}}$$ (29)

and the † denotes the transposed, complex-conjugated matrix.

The linear response equations, Eqs. (22) and (23), can be written, using this notation, as

$$(H-\hbar \omega \sigma_{3})\psi (r)=-\sigma_{3}g(r),$$ (30)

where

$$\psi (r)=\left(\begin{array}{c} u(r)\\ \upsilon (r) \end{array}\right),\;g(r)=\left(\begin{array}{c} N_{0}^{1/2}f_{+}(r)\psi_{g}(r)\\ -N_{0}^{1/2}f_{-}^{*}(r)\psi_{g}^{*}(r) \end{array}\right).$$ (31)

The solution of the linear response equations is found by expanding both *ψ*(***r***) and *g*(***r***) in the normal modes

$$\psi (r)=\sum_{\lambda }c_{\lambda }\phi_{\lambda }(r),\;g(r)=\sum_{\lambda }g_{\lambda }\phi_{\lambda }(r).$$ (32)

Where the *g*_λ_ are given by the following overlap integral

$$g_{\lambda }=\int dr\phi_{\lambda }^{†}(r)\sigma_{3}g(r).$$ (33)

Substituting these expansions into Eq. (30) yields a system of completely uncoupled equations to be solved for the *c*_λ_. The final solution is written as

$$\psi (r)=-\sum_{\lambda }\frac{\left(g_{\lambda }/\hbar \right)}{\omega_{\lambda }-\omega }\phi_{\lambda }(r).$$ (34)

Note that the linear response diverges when the condensate is driven exactly on resonance. This unphysical behavior results from our neglect of loss processes and nonlinear effects.

## 3. Mean-Field Theory: Comparison with Experiment

Mean-field theory provides predictions for a variety of measurable condensate properties. These properties include condensate geometries, densities, and excitation frequencies. In this section, we shall discuss the comparison of the predicted values of these quantities with experiment. Measurements of condensate properties have been, to date, primarily performed on condensates formed in the traps of Refs. [1] and [3]. Comparisons with properties of condensates formed in the trap of Ref. [2] are not possible at present because measurements of these properties still have substantial uncertainties.

The geometry, density, and excitation frequency predictions of the MFT equations do not contain any adjustable parameters. The numerical constants that are input into the theory are the atomic mass (*M*), the radial and axial trap frequencies (*ω_r_* and ω*_z_*), the number of condensate atoms (*N*_0_), and the scattering length (*a*). All of these numbers are determined experimentally. The scattering length, in particular, is determined principally by photoassociation spectroscopic measurements [14,21].

Condensate lifetimes, on the other hand, depend critically on two- and three-body scattering event rates. These rates are quite difficult to determine accurately [13]. Therefore we will not consider lifetimes in the comparison of MFT with experiment.

### 3.1. Geometries and Densities

#### 3.1.1

Geometries and densities were among the first condensate properties to be measured (see e.g., Ref. [1]). It is important to note that both of these ^87^Rb condensate properties are determined indirectly. That is, after condensate formation, the trap potential is dramatically lowered, allowing the condensate to undergo a ballistic expansion, and then an absorption picture is taken. This picture, which essentially exhibits the velocity distribution of the original condensate, can then be used to extrapolate back to the original spatial density distribution.

The most quantitative comparison of theoretical and experimental geometry and density performed to date is that of Holland and Cooper (Ref. [22]). In this work, the time-dependent GP equation was solved by direct numerical integration. The time variations of the trap potential that occurred in the experiment of Ref. [1] were modeled and a prediction for the absorption picture was obtained. The agreement between theory and experiment for the velocity distribution appears to be at the 5 % level.

**Fig. 1:**
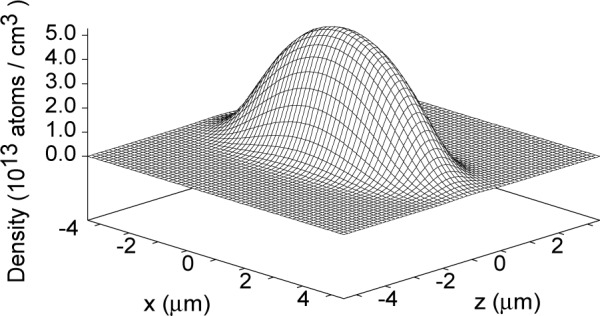
A plot of the spatial distribution of the ground state ^87^Rb condensate density over a plane containing the *z*-axis. In this plot, *N*_0_ = 2012 atoms.

**Fig. 2:**
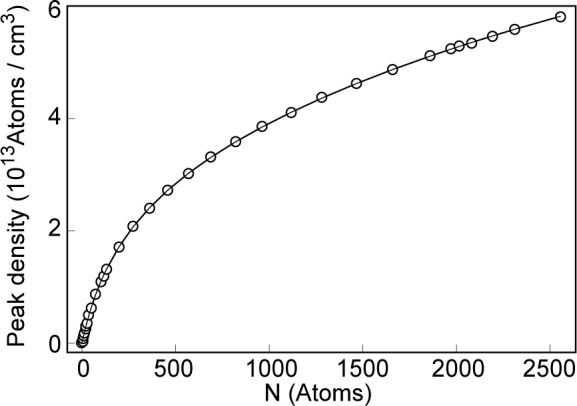
A plot of the peak condensate density as a function of condensate population for a ^87^Rb condensate confined in the strong TOP trap.

To date, no measurements of condensate geometries and densities of the ^87^Rb condensate have been obtained *in situ*. However, it is interesting to see what the MFT predictions are for this case. Figure 1 shows the density profile for the ^87^Rb condensate for the trap parameters of Ref. [1] for the case of *N*_0_ = 2012 atoms. A plot of peak density as a function of condensate population is shown in Fig. 2. The peak density for this case is 5. 2 × 10^13^ atoms/cm^3^. The extrapolated experimental result is 3 × 10^13^ atoms/cm^3^ but this number is accompanied by substantial error bars within which the MFT number falls.

#### 3.1.2 ^23^Na

To date, condensates have been formed in two different traps at MIT. In the optical-plug trap, atoms were prevented from undergoing majorana spin flips through the use of a blue laser focussed at the center of the trap [3]. The “cloverleaf” trap is an Ioffe-Pritchard trap and thus there is no zero of the magnetic field [23]. The condensates formed in each of these traps are very different from the ^87^Rb one. The major difference is size—the ^23^Na condensates contain on the order of 10^6^ atoms.

Mean-field theory solutions for condensates of this size can be obtained via the “Thomas-Fermi” approximation [24]. The approximation amounts to the neglect of the kinetic energy term in the Gross-Pitaevskii equation. This reduces Eq. (8) to

$$(V_{\mathrm{trap}}(r)+N_{0}U_{0}{\left|\psi (r)\right|}^{2}\psi (r)=\mu \psi (r),$$ (35)

where the kinetic energy term, − (*ħ*^2^/2*M*)=∇^2^*ψ*(*r*), has been neglected. The wavefunction thus has the form

$$\psi (r)=\{\begin{array}{ll} {\left[\frac{\mu -V_{\mathrm{trap}}(r)}{N_{0}U_{0}}\right]}^{1/2}, & \text{for}\;V_{\mathrm{trap}}(r)<\mu ;\\ 0, & \text{for}\;V_{\mathrm{trap}}(r)\geq \mu , \end{array}$$ (36)

The relationship between *N*_0_ and *μ* is found by the normalization condition

$$\int dr{\left|\psi (r)\right|}^{2}=1.$$ (37)

**Fig 3:**
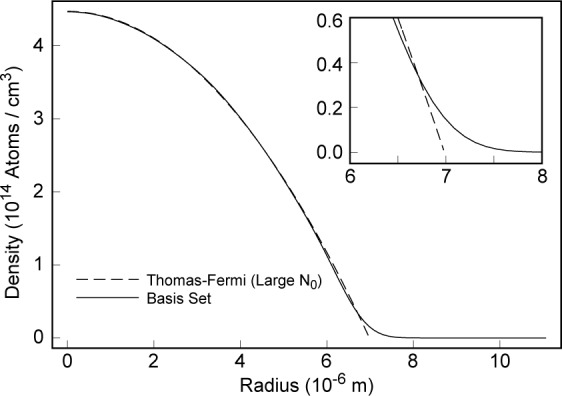
Comparison of the Thomas-Fermi and MFT solutions for the MIT condensates. The condensate consists of 5 million ^23^Na atoms confined in a cylindrically symmetric trap with *ν_r_* = 350 Hz and *ν_z_* = 18 Hz. The basis-set calculation required 350 functions. The plot shows the condensate density in the *z* = 0 plane. Note that the correction to the Thomas-Fermi result (shown inset) is important only at the very edge of the condensate.

A comparison of the Thomas-Fermi approximate solution with the basis-set solution is shown in Fig. 3.

The comparison of the geometries and densities of the ^23^Na condensates with the results of MFT suffers from the uncertainty in the value of the sodium scattering length. In contrast to the ^87^Rb scattering length, until recently the ^23^Na scattering length was known only to within a factor of two [14]. The MFT solutions depend only on the parameter *N*_0_*a*/*l*_sho_ where *l*_sho_ is the harmonic oscillator length scale. Uncertainty in *a* will lead to a similar uncertainty in MFT predictions.

### 3.2 Excitations

**Fig. 4:**
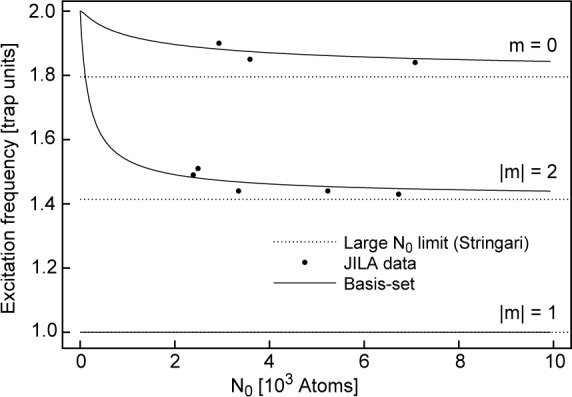
Comparison of the JILA excitations results with MFT predictions. This graph is reprinted from Edwards et al., Phys. Rev. Lett. 77, 1671 (1996).

The excitation data provide the opportunity for the most quantitative comparison with MFT to date. Excitation frequencies are measured by forming a condensate, driving it weakly by oscillating the trap potential, waiting a specified delay time, and then probing the condensate. This cycle was repeated for increasing delay times forming a time history of condensate oscillations. The width of the condensate was observed to oscillate and the frequency of this oscillation measured. This experiment was recently performed both at JILA [25] and at MIT [26]. The comparison [27] with the JILA results is shown in Fig. 4. The agreement varies between 2 % and 5 %. Similar agreement with the theory [28] was found in the MIT experiment.

### 3.3 Summary

This paper has been written in the early days of quantitative modelling of dilute atomic BECs, and there have yet been relatively few stringent tests of the validity of MFT. However, it has been found to have good predictive and interpretive value. As the accuracy of the experiments improves, and the uncertainties in the values of the microscopic parameters are reduced, we expect that dilute atomic BECs will provide a new testing ground for the Bogoliubov approximation and its variants, which are among the cornerstones of the quantum theory of many-particle systems.

## 4. Appendix A. Numerical Techniques

### 4.1 Solving the Gross-Pitaevskii Equation

The time-independent GP equation is written

$$\left(-\frac{\hbar^{2}}{2M}\nabla^{2}+V_{\mathrm{trap}}(r)+N_{0}U_{0}{\left|\psi (r)\right|}^{2}\right)\psi (r)=\mu \psi (r).$$ (38)

We expand *ψ*(***r***) in the basis of trap-potential eigenfunctions, i.e.,

$$\psi (r)=\sum_{\lambda }a_{\lambda }\phi_{\lambda }(r),$$ (39)

where the sum extends over the *N*_basis_ basis-set functions, λ represents a set of quantum numbers, and *ϕ*_λ_(***r***) is an eigenfunction of the trap Hamiltonian,

$$H_{0}\phi_{\lambda }(r)=\left\{-\frac{\hbar^{2}}{2M}\nabla^{2}+V_{\mathrm{trap}}(r)\right\}\phi_{\lambda }(r)$$ (40)

$$=\varepsilon_{\lambda }\phi_{\lambda }(r).$$ (41)

Inserting Eq. (39) into Eq. (38) and taking the scalar product with each basis-set function converts the problem to one of finding the simultaneous root of *N*_basis_ nonlinear algebraic functions having the following form

$$f_{\lambda }(\{a\})\equiv (\varepsilon_{\lambda }-\mu )a_{\lambda }+N_{0}U_{0}\sum_{\lambda_{1},\lambda_{2},\lambda_{3}}C(\lambda ,\lambda_{1},\lambda_{2},\lambda_{3})a_{\lambda_{1}}^{*}a_{\lambda_{2}}a_{\lambda_{3}}=0.$$ (42)

The coefficients *C*(λ_1_, λ_2_, λ_3_, λ_4_) are the integrals of the product of four trap wavefunctions; several methods for evaluating these integrals are described in Appendix 5. These nonlinear equations can be solved by a standard Newton-Raphson [29] technique provided a good guess at the solution is known.

Equations (42) are not sufficient to determine the set {*a*} since *μ* is also an unknown. If we choose a value of *μ*, and a corresponding value of *N*_0_, a solution may be found that will, in general, not be normalized. This solution may be normalized by the simultaneous rescaling of the basis-set coefficients {*a*}, and the number of condensate atoms, *N*_0_.

For example, find an arbitrary solution $\psi_{N_{A}}(r)$ of the time independent GP equation

$$\left(-\frac{\hbar^{2}}{2M}\nabla^{2}+V_{\mathrm{trap}}(r)+N_{A}U_{0}{\left|\psi_{N_{A}}(r)\right|}^{2}\right)\psi_{N_{A}}(r)=\mu \psi_{N_{A}}(r),$$ (43)

then find the norm of the solution

$$A^{2}=\int dr{\left|\psi_{N_{A}}(r)\right|}^{2},$$ (44)

where *A* is some constant. The set {*a_A_*}, the basis-set coefficients for the solution $\psi_{N_{A}}(r)$, and the condensate population *N_A_* may be rescaled as follows

$$\{a_{B}\}=\left\{\frac{a_{A}}{A}\right\},\;\text{and}\;N_{B}=A^{2}N_{A},$$ (45)

to give a normalized solution $\psi_{N_{A}}(r)$ for the new GP equation

$$\left(-\frac{\hbar^{2}}{2M}\nabla^{2}+V_{\mathrm{trap}}(r)+N_{B}U_{0}{\left|\psi_{N_{B}}(r)\right|}^{2}\right)\psi_{N_{B}}(r)=\mu \psi_{N_{B}}(r).$$ (46)

So we find an arbitrary solution $\psi_{N_{A}}(r)$, then find a normalized solution $\psi_{N_{B}}(r)$ from $\psi_{N_{A}}(r)$ by rescaling both the wavefunction and the number of atoms in the condensate. This method is also discussed in Refs. [30,31].

To ensure that the final normalized solution is actually the lowest energy state of the condensate, we simulate the adiabatic growth of the nonlinear term in Eq. (38) by first finding a solution for a value of *μ* close to the noninteracting ground state energy, which corresponds to a very small number of particles in the condensate, and so the condensate wavefunction is very close to that of the trap ground state. The initial values of the basis-set coefficients are those of the trap ground state, i.e., {a} ={1,0,0, …}. The Newton-Raphson algorithm is then used to solve Eqs. (42). The chemical potential *μ* is then incremented, corresponding to a increase in condensate size, and the basis-set coefficients found previously, for the smaller condensate, are used as the initial values for this new solution. This process, which is repeated for increasing values of *μ*, gives not only the lowest energy solution, but also generates solutions for a large range of *μ*, and corresponding *N*_0_, with little wasted computational time.

### 4.2 Solving the Bogoliubov Equations

In this section we describe how Eqs. (13) and (14) are numerically solved using a finite basis consisting of trap eigenfunctions. The solution is found by first expanding *u*_λ_(***r***) and *υ*_λ_(***r***), in the eigenstates of *H*_0_ to produce a set of algebraic equations. These equations are then cast into a generalized matrix eigenvalue equation having the form *A****x***_λ_= *w*_λ_*B****x***_λ_. In this equation, *A* and *B* are square matrices of order 2*N*_basis_ (where *N*_basis_ denotes the number of basis-set states used), ***x***_λ_ is an 2*N*_basis_-component column vector containing the coefficients in the basis-set expansion of *u*_λ_(***r***) and *υ*_λ_ (***r***), and *w*_λ_ is the generalized eigenvalue. We shall treat the case of a spherically symmetric harmonic trap potential for simplicity. It is important when solving the normal-mode equations using a finite basis that Eq. (21) for *ψ*_g_(*r*) also be solved within this set.

The exact expansion of *u*_λ_(***r***) and *υ*_λ_(***r***) in eigenfunctions of *H*_0_ has the form

$$u_{\lambda }(r)=\sum_{s\prime ,l\prime ,m\prime }a_{s\prime l\prime m\prime }^{\left(\lambda \right)}\psi_{2s\prime }+l\prime ,l\prime ,m\prime (r)$$ (47)

and

$$\upsilon_{\lambda }(r)=\sum_{s\prime ,l\prime ,m\prime }b_{s\prime l\prime m\prime }^{\left(\lambda \right)}\psi_{2s\prime }+l\prime ,l\prime ,m\prime (r)$$ (48)

where

$$H_{0}{\psi_{2}}_{s+l,l,m}(r)=(2s+l+3/2)\hbar \omega_{\mathrm{trap}}\psi_{2s+l,l,m}(r)\equiv \varepsilon_{s}^{\left(lm\right)}\hbar \omega_{\mathrm{trap}}\psi_{2s+l,l,m}(r)$$ (49)

defines the eigenvalues and eigenfunctions of *H*_sho_. The ranges of variation of the quantum numbers are *s*, *l* = 0, 1, … and *m* = −*l*, …, *l*.

Truncating and inserting these expansions into Eqs. (24) and (25) and projecting each equation onto an arbitrary eigenstate of *H*_0_ yields the following system of linear equations

$$(\varepsilon_{s}^{\left(lm\right)}–(\beta +\alpha_{\lambda }))a_{slm}^{\left(\lambda \right)}+2\sum_{\;s\prime =0}^{N_{\mathrm{basis}}–1}w_{ss\prime }^{\left(lm\right)}a_{s^{\prime }lm}^{\left(\lambda \right)}+\sum_{\;s\prime =0}^{N_{\mathrm{basis}}–1}w_{ss\prime }^{\left(lm\right)}b_{s^{\prime }lm}^{\left(\lambda \right)}=0$$ (50)

and

$$(\varepsilon_{s}^{\left(lm\right)}–(\beta -\alpha_{\lambda }))b_{slm}^{\left(\lambda \right)}+\sum_{\;s\prime =0}^{N_{\mathrm{basis}}–1}w_{ss\prime }^{\left(lm\right)}a_{s^{\prime }lm}^{\left(\lambda \right)}+2\sum_{\;s\prime =0}^{N_{\mathrm{basis}}–1}w_{ss\prime }^{\left(lm\right)}b_{s^{\prime }lm}^{\left(\lambda \right)}=0.$$ (51)

where *μ* = *βħ*ω_trap_ and ω_λ_ = α_λ_ω_trap_. The full set of equations that must be solved to find the a*_l_* consists of those given just above for all values of λ and *μ*. The matrix element $w_{ss\prime }^{\left(lm\right)}$ is given by

$$w_{ss\prime }^{\left(lm\right)}\equiv \frac{1}{\hbar \omega_{\mathrm{trap}}}\int_{0}^{\infty }dru_{sl}(r)\left[N_{0}U_{0}{\left|\psi_{g}(r)\right|}^{2}\right]u_{s^{\prime }l}(r).$$ (52)

where $\psi_{2s+l,l,m}(r)=u_{sl}(r)Y_{lm}(\hat{r})/r$ defines the reduced radial wavefunction *u_sl_*(*r*).

The spherical symmetry of the condensate ground state imposes a particularly simple structure upon the equations for $a_{slm}^{\left(\lambda \right)}$ and $b_{slm}^{\left(\lambda \right)}$. These equations divide into subsets of equations that are self-contained within the subspace of fixed angular momentum and magnetic quantum number, i.e., there is no coupling between $a_{slm}^{\left(\lambda \right)}$ or $b_{slm}^{\left(\lambda \right)}$ and either $a_{sl^{\prime }m^{\prime }}^{\left(\lambda \right)}$ or $b_{sl^{\prime }m^{\prime }}^{\left(\lambda \right)}$. Thus an infinite number of normal-mode solutions, each proportional to a single *Y_lm_*, can be found by setting to zero the *a* and *b* coefficients corresponding to all pairs (*l*′, *m*′) *except one* [say (*l*, *m*)] in expansion Eqs. (47) and (48). We can replace the eigenvalue label *λ* with the quantum numbers *l* and *m* plus a “radial” quantum number *n*. The significance of this quantum number can be understood by writing the normal modes in terms of their basis-set expansions with the above symmetries imposed

$$u_{nlm}(r)=\frac{1}{r}Y_{lm}(\hat{r})\sum_{s=0}^{N_{\mathrm{basis}}-1}a_{slm}^{\left(n\right)}u_{sl}(r)\equiv U_{nlm}(r)Y_{lm}(\hat{r})$$ (53)

and

$$\upsilon_{nlm}(r)=\frac{1}{r}Y_{lm}(\hat{r})\sum_{s=0}^{N_{\mathrm{basis}}-1}b_{slm}^{\left(n\right)}u_{sl}(r)\equiv V_{nlm}(r)Y_{lm}(\hat{r}).$$ (54)

The quantum number *n* is defined as the number of nodes found in the radial wavefunctions, *U_nlm_*(*r*) and *V_nlm_*(*r*). Furthermore, *n* runs from − ∞ to ∞ because the normal frequencies occur in pairs of opposite sign. That this must be the case can be understood by examining Eqs. (24) and (25). If ω_λ_ → − ω_λ_ and if *u*_λ_ and *υ*_λ_ are exchanged, then Eqs. (24) and (25) remain unchanged. Hence, if there exists a solution of the normal-mode equations for positive ω_λ_, then a negative frequency solution also exists having the roles of *u*_λ_ and *υ*_λ_ reversed. Indeed, we find that normal frequencies occur in equal-magnitude pairs of opposite sign in our solutions. A negative value of *n*, therefore, labels a normal-mode solution obtained by performing the above transformation on the corresponding positive-*n* solution. The *n* = *l* = *m* = 0 normal-mode solution deserves special comment. This solution always has a frequency of zero (i.e., *ω*_000_ = 0) with *u*_000_(***r***) and *υ*_000_(***r***) being proportional to *ψ*_g_(*r*). This *condensate-mode solution* has zero norm, see Eq. (29).

Moving the terms in Eqs. (50) and (51) containing *α*_λ_to the right-hand-side gives

$$(\varepsilon_{s}^{\left(lm\right)}–\beta )a_{slm}^{\left(n\right)}+2\sum_{\;s\prime =0}^{N_{\mathrm{basis}}–1}w_{ss\prime }^{\left(lm\right)}a_{s^{\prime }lm}^{\left(n\right)}+\sum_{\;s\prime =0}^{N_{\mathrm{basis}}–1}w_{\mathrm{ss}\prime }^{\left(lm\right)}b_{s^{\prime }lm}^{\left(n\right)}=\alpha_{nlm}a_{slm}^{\left(n\right)}$$ (55)

and

$$(\varepsilon_{s}^{\left(lm\right)}–\beta )b_{slm}^{\left(n\right)}+\sum_{\;s\prime =0}^{N_{\mathrm{basis}}–1}w_{ss\prime }^{\left(lm\right)}a_{s^{\prime }lm}^{\left(n\right)}+2\sum_{\;s\prime =0}^{N_{\mathrm{basis}}–1}w_{ss\prime }^{\left(lm\right)}b_{s^{\prime }lm}^{\left(n\right)}=-\alpha_{nlm}b_{slm}^{\left(n\right)}$$ (56)

where we have replaced *α*_λ_ with *α_nlm_* and ( $a_{s\prime lm}^{\left(\lambda \right)}$, $b_{s\prime lm}^{\left(\lambda \right)}$) with $a_{s\prime lm}^{\left(\lambda \right)}$, $b_{s\prime lm}^{\left(\lambda \right)}$. The above *N*_basis_ pairs of equations can be summarized as a single (2 × 2) matrix equation by making the following definitions.

$$c_{s}^{nlm}=\left(\begin{array}{c} a_{slm}^{n}\\ b_{slm}^{n} \end{array}\right),\;\sigma =\left(\begin{array}{cc} 2 & 1\\ 1 & 2 \end{array}\right),\;\sigma_{3}\left(\begin{array}{cc} 1 & 0\\ 0 & -1 \end{array}\right),$$ (57)

With these definitions, Eqs. (55) and (56) can be written as

$$(\varepsilon_{s}^{\left(lm\right)}–\beta )I_{2}C_{s}^{\left(nlm\right)}+\sum_{\;s\prime =0}^{N_{\mathrm{basis}}–1}w_{ss\prime }^{\left(lm\right)}\sigma c_{s\prime }^{\left(nlm\right)}=\alpha_{nlm}\sigma_{3}c_{s}^{\left(nlm\right)}$$ (58)

where *I*_2_ is the (2 × 2) unit matrix and where s runs from 0 to *N*_basis_ −1.

The final transition to the form of a generalized eigenvalue problem is accomplished by defining the following (*N*_basis_ × *N*_basis_) matrices

$${\left(d^{\left(lm\right)}\right)}_{ss\prime }=(\varepsilon_{s}^{\left(lm\right)}–\beta )\delta_{ss\prime },$$ (59)

and

$${\left(w^{\left(lm\right)}\right)}_{ss\prime }=w_{ss\prime }^{\left(lm\right)}.$$ (60)

Using these matrices, Eq. (58) may be written as a single matrix equation

$$A^{\left(lm\right)}c^{\left(nlm\right)}=\alpha_{nlm}B^{\left(lm\right)}c^{\left(nlm\right)},$$ (61)

where ***c***^(^*^nlm^*^)^ is given by

$$c^{\left(nlm\right)}=\left(\begin{array}{c} {c_{0}}^{\left(nlm\right)}\\ .\\ .\\ .\\ {c_{N}}_{{\mathrm{basis}}^{-1}}^{\left(nlm\right)} \end{array}\right)$$ (62)

and where the matrix *A*^(^*^lm^*^)^ can be expressed as a tensor product of matrices:

$$A^{\left(lm\right)}=d^{\left(lm\right)}⊗I_{2}+w^{\left(lm\right)}⊗\sigma$$ (63)

and *B* is given by

$$B^{\left(lm\right)}=I_{N_{\mathrm{basis}}}⊗\sigma_{3}.$$ (64)

where $I_{N_{\mathrm{basis}}}$ is the unit matrix of order *N*_basis_.

The square matrix on the left of the ⊗ symbol is of order *N*_basis_ and the matrix on its right is of order 2. The full matrix is of order 2*N*_basis_ with elements that can be indexed as *C*_(_*_s,k_*_)(_*_s'k'_*_)_ = *A_ss_*_'_*B_kk'_*, where *s* runs from 0 to *N*_basis_ − 1 and *k* runs over 0, 1.

The component expressions for the matrices appearing in the final generalized-eigenvalue problem are as follows

$${\left(A^{\left(lm\right)}\right)}_{\left(s,k)(s^{\prime }k^{\prime }\right)}=\;{\left(d^{\left(lm\right)}\right)}_{ss\prime }\delta_{kk^{\prime }}+\;{\left(w^{\left(lm\right)}\right)}_{ss\prime }(1+\delta_{kk^{\prime }})$$ (65)

and

$$(B^{\left(lm\right)}{)}_{\left(s,k)(s^{\prime }k^{\prime }\right)}=\delta_{ss\prime }\delta_{kk^{\prime }}(\delta_{k0}–\delta_{k1}).$$ (66)

Furthermore,

$$c_{\left(s,0\right)}^{\left(nlm\right)}={a^{\left(n\right)}}_{slm},\;c_{\left(s,1\right)}^{\left(nlm\right)}={b^{\left(n\right)}}_{slm}.$$ (67)

## 5. Appendix B. Integration Methods

The basis-set expansion method requires the evaluation of a set of integrals, the integrals of four basis-set functions over all space. In general the basis-set functions would be those for an anisotropic trapping potential, though in two of the cases of interest [1,2] they are eigenfunctions of the cylindrically symmetric harmonic oscillator. We will now consider how these integrals may be evaluated.

### 5.1 Cylindrical Symmetric Traps

The integral which must be evaluated is given by

$$\begin{array}{l} C\left(\lambda_{1},\lambda_{2},\lambda_{3},\lambda_{4}\right)=\int_{0}^{2\pi }d\phi \int_{0}^{\infty }d\rho \rho \\ \int_{-\infty }^{\infty }dz{\phi^{*}}_{\lambda_{1}}\left(r\right){\phi^{*}}_{\lambda_{2}}\left(r\right)\phi_{\lambda_{3}}\left(r\right)\phi_{\lambda_{4}}\left(r\right) \end{array}$$ (68)

where the basis-set functions *ϕ_λ_* (***r***), for a harmonic trapping potential

$$V_{\mathrm{trap}}\left(r\right)=M\left({\omega_{\rho }}^{2}\rho^{2}+{\omega_{z}}^{2}z^{2}\right)/2,$$ (69)

are given by

$$\phi_{\lambda_{i}}\left(r\right)={\left[\frac{\alpha_{z}}{\pi^{\frac{1}{2}}2^{n_{z_{i}}}n_{z_{i}}!}\right]}^{\frac{1}{2}}e^{-\frac{1}{2}{\alpha^{2}}_{z}z^{2}}H_{n_{z_{i}}}\left(\alpha_{z}z\right)\times {\left[\frac{\alpha_{\rho }^{2}n_{\rho_{i}}!}{\pi \left(n_{\rho_{i}}+m_{i}\right)!}\right]}^{\frac{1}{2}}e^{im_{i}\phi }{\left(\alpha_{\rho }\rho \right)}^{m_{i}}e^{-\frac{1}{2}{\alpha^{2}}_{\rho }\rho^{2}}L_{n_{\rho_{i}}}^{\left(m_{i}\right)}\left({\alpha^{2}}_{\rho }\rho^{2}\right),$$ (70)

where *ρ* = (*x* ^2^ + *y* ^2^)^1/2^ and *α_ρ,z_* = (*Mω_ρ,z_*/*ℏ*)^1/2^. These functions may be rewritten, with substitutions *ζ* = (*α_ρ_ρ*)^2^ and *ξ* = *α_z_z*, to give

$$\phi_{\lambda_{i}}\left(r\right)=N_{\lambda_{i}}e^{-\frac{1}{2}\xi^{2}}H_{n_{z_{i}}}\left(\xi \right)e^{im_{i\phi }}\zeta^{\frac{m_{i}}{2}}e^{-\frac{1}{2}\zeta }L_{n_{\rho_{i}}}^{\left(m_{i}\right)}\left(\zeta \right),$$ (71)

the normalization constant remains unchanged,

$$N_{\lambda_{i}}={\left[\left(\frac{\alpha_{z}}{\pi^{\frac{1}{2}}2^{n_{z_{i}}}n_{z_{i}}!}\right)\left(\frac{\alpha_{\rho }^{2}n_{\rho_{i}}!}{\pi \left(n_{\rho_{i}}+m_{i}\right)!}\right)\right]}^{\frac{1}{2}}$$ (72)

and the integral operator transforms,

$$\int_{0}^{2\pi }d\phi \int_{0}^{\infty }d\rho \rho \int_{-\infty }^{\infty }dz\to \frac{1}{2\alpha_{z}\alpha_{\rho }^{2}}\int_{0}^{2\pi }d\phi \int_{0}^{\infty }d\zeta \int_{-\infty }^{\infty }d\xi .$$ (73)

These substitutions reduce Eq. (68) to

$$\begin{array}{c} C\left(\lambda_{1},\lambda_{2},\lambda_{3},\lambda_{4}\right)=\frac{N_{\lambda_{1}}N_{\lambda_{2}}N_{\lambda_{3}}N_{\lambda_{4}}}{2\alpha_{\rho }^{2}\alpha_{z}}\int_{0}^{2\pi }d\phi \;e^{i\phi \left(m_{3}+m_{4}-m_{1}-m_{2}\right)}\\ \times \int_{0}^{\infty }d\zeta \;e^{-2\zeta }\zeta^{\frac{1}{2}\left(m_{1}+m_{2}+m_{3}+m_{4}\right)}L_{n_{\rho_{1}}}^{\left(m_{1}\right)}\left(\zeta \right)\;L_{n_{\rho_{2}}}^{\left(m_{2}\right)}\left(\zeta \right)\\ \times L_{n_{\rho_{3}}}^{\left(m_{3}\right)}\left(\zeta \right)L_{n_{\rho_{4}}}^{\left(m_{4}\right)}\left(\zeta \right)\\ \int_{-\infty }^{\infty }d\xi e^{-2\xi^{2}}H_{n_{z_{1}}}\left(\xi \right)H_{n_{z_{2}}}\left(\xi \right)H_{n_{z_{3}}}\left(\xi \right)H_{n_{z_{4}}}\left(\xi \right), \end{array}$$ (74)

so the integral has been broken down into three one-dimensional integrals, i.e.,

$$\begin{array}{l} C\left(\lambda_{1},\lambda_{2},\lambda_{3},\lambda_{4}\right)=\frac{N_{\lambda_{1}}N_{\lambda_{2}}N_{\lambda_{3}}N_{\lambda_{4}}}{2\alpha_{\rho }^{2}\alpha_{z}}\Phi \left(\lambda_{1},\lambda_{2},\lambda_{3},\lambda_{4}\right)\\ \times U\left(\lambda_{1},\lambda_{2},\lambda_{3},\lambda_{4}\right)\Xi \left(n_{z_{1}},n_{z_{2}},n_{z_{3}},n_{z_{4}}\right) \end{array}$$ (75)

where

$$\begin{array}{l} \Phi \left(\lambda_{1},\lambda_{2},\lambda_{3},\lambda_{4}\right)=\int_{0}^{2\pi }d\phi e^{i\phi \left(m_{3}+m_{4}-m_{1}-m_{2}\right)},\\ U\left(\lambda_{1},\lambda_{2},\lambda_{3},\lambda_{4}\right)=\int_{0}^{\infty }d\zeta e^{-2\zeta }\zeta^{\frac{1}{2}\left(m_{1}+m_{2}+m_{3}+m_{4}\right)} \end{array}$$ (76)

$$\times L_{n_{\rho_{1}}}^{\left(m_{1}\right)}\left(\zeta \right)L_{n_{\rho_{2}}}^{\left(m_{2}\right)}\left(\zeta \right)L_{n_{\rho_{3}}}^{\left(m_{3}\right)}\left(\zeta \right)L_{n_{\rho_{4}}}^{\left(m_{4}\right)}\left(\zeta \right)$$ (77)

and

$$\Xi \left(n_{z_{1}},n_{z_{2}},n_{z_{3}},n_{z_{4}}\right)=\int_{-\infty }^{\infty }d\xi e^{-2\xi^{2}}H_{n_{z_{1}}}\left(\xi \right)H_{n_{z_{2}}}\left(\xi \right)H_{n_{z_{3}}}\left(\xi \right)H_{n_{z_{4}}}\left(\xi \right).$$ (78)

### 5.2 Completely Anisotropic Traps

The integral to be evaluated for a completely anisotropic trap,

$$V_{\mathrm{trap}}\left(r\right)=M\left(\omega_{x}^{2}x^{2}+\omega_{y}^{2}y^{2}+\omega_{y}^{2}z^{2}\right)/2,$$ (79)

is given by

$$A\left(\lambda_{1},\lambda_{2},\lambda_{3},\lambda_{4}\right)=\int_{-\infty }^{\infty }dx\int_{-\infty }^{\infty }dy\int_{-\infty }^{\infty }dz\phi_{\lambda_{1}}^{*}\left(r\right)\phi_{\lambda_{2}}^{*}\left(r\right)\phi_{\lambda_{3}}\left(r\right)\phi_{\lambda_{4}}\left(r\right)$$ (80)

where the basis-set functions *ϕ_λ_* (***r***) are now given by:

$$\begin{array}{c} \phi_{\lambda i}\left(r\right)={\left[\frac{\alpha_{x}}{\pi^{\frac{1}{2}}2^{n_{x_{i}}}n_{x_{i}}!}\right]}^{\frac{1}{2}}e^{-\frac{1}{2}\alpha_{x}^{2}x^{2}}H_{n_{x_{i}}}\left(\alpha_{x}x\right)\\ \times {\left[\frac{\alpha_{y}}{\pi^{\frac{1}{2}}2^{n_{y_{i}}}n_{y_{i}}!}\right]}^{\frac{1}{2}}e^{-\frac{1}{2}\alpha_{y}^{2}y^{2}}H_{n_{y_{i}}}\left(\alpha_{y}y\right)\\ \times {\left[\frac{\alpha_{z}}{\pi^{\frac{1}{2}}2^{n_{z_{i}}}n_{z_{i}}!}\right]}^{\frac{1}{2}}e^{-\frac{1}{2}\alpha_{z}^{2}z^{2}}H_{n_{z_{i}}}\left(\alpha_{z}z\right) \end{array}$$ (81)

where *α_x,y,z_* = (*Mω_x,y,z_*/*ℏ*)^1/2^. These functions may be written,

$$\phi_{\lambda_{i}}\left(r\right)=N_{\lambda_{i}}e^{-\left(\alpha_{x}^{2}x^{2}+\alpha_{y}^{2}y^{2}+\alpha_{z}^{2}z^{2}\right)/2}\times H_{n_{x_{i}}}\left(\alpha_{x}x\right)H_{n_{y_{i}}}\left(\alpha_{y}y\right)H_{n_{z_{i}}}\left(\alpha_{z}z\right).$$ (82)

With the substitutions *α_x_x* = *u*, *α_y_y* = *υ* and *α_z_z* = *w*, this reduces Eq. (80) to

$$\begin{array}{l} A\left(\lambda_{1},\lambda_{2},\lambda_{3},\lambda_{4}\right)=\frac{N_{\lambda_{1}}N_{\lambda_{2}}N_{\lambda_{3}}N_{\lambda_{4}}}{\alpha_{x}\alpha_{y}\alpha_{z}}\\ \times \int_{-\infty }^{\infty }du\;e^{-2u^{2}}H_{n_{z_{1}}}\left(u\right)H_{n_{z_{2}}}\left(u\right)H_{n_{z_{3}}}\left(u\right)H_{n_{z_{4}}}\left(u\right)\\ \times \int_{-\infty }^{\infty }d\upsilon \;e^{-2\upsilon^{2}}H_{n_{z_{1}}}\left(\upsilon \right)H_{n_{z_{2}}}\left(\upsilon \right)H_{n_{z_{3}}}\left(\upsilon \right)H_{n_{z_{4}}}\left(\upsilon \right)\\ \times \int_{-\infty }^{\infty }dw\;e^{-2w^{2}}H_{n_{z_{1}}}\left(w\right)H_{n_{z_{2}}}\left(w\right)H_{n_{z_{3}}}\left(w\right)H_{n_{z_{4}}}\left(w\right) \end{array}$$ (83)

$$A\left(\lambda_{1},\lambda_{2},\lambda_{3},\lambda_{4}\right)=\frac{N_{\lambda_{1}}N_{\lambda_{2}}N_{\lambda_{3}}N_{\lambda_{4}}}{\alpha_{x}\alpha_{y}\alpha_{z}}\times \Xi \left(n_{x_{1}},n_{x_{2}},n_{x_{3}},n_{x_{4}}\right)\Xi \left(n_{y_{1}},n_{y_{2}},n_{y_{3}},n_{y_{4}}\right)\times \Xi \left(n_{z_{1}},n_{z_{2}},n_{z_{3}},n_{z_{4}}\right)$$ (84)

we will now outline some of the methods we may use to evaluate these integrals.

### 5.3 Exact and Series Methods

Equation (76) may be evaluated easily to give

$$\Phi \left(\lambda_{1},\lambda_{2},\lambda_{3},\lambda_{4}\right)=\{\begin{array}{ll} 2\pi , & \text{for}\;m_{1}+m_{2}=m_{3}+m_{4};\\ 0, & \text{otherwise}. \end{array}$$ (85)

Equation (78) may be evaluated by use of an addition formula for Hermite polynomials [32] to give

$$\Xi \left(n_{1},n_{2},n_{3},n_{4}\right)=\{\begin{array}{ll} \frac{n_{3}!n_{4}!}{\pi } & \sum_{t=0}^{\min\left(n_{3},n_{4}\right)}\frac{2^{t+s-1}\Gamma \left(s-n_{1}\right)\Gamma \left(s-n_{2}\right)}{t!\left(n_{3}-t\right)!\left(n_{4}-t\right)!}\\ & \times \Gamma \left(s-n_{3}-n_{4}+2t\right),\\ & \text{if}\;n_{1}+n_{2}+n_{3}+n_{4}\;\text{is}\;\text{even};\\ 0, & \text{otherwise}. \end{array}$$ (86)

where *s* = (*n*_1_+*n*_2_+*n*_3_+*n*_4_−2*t*+1)/2.

By expanding the Laguerre polynomials in Eq. (77) as finite series [33], an even more complicated expression can be derived for the cylindrically symmetric case. While these results are exact, they are of little practical use for computational calculations in standard extended precision arithmetic, since numerical accuracy is rapidly lost in the summations of terms which vary over several orders of magnitude. An alternative method for evaluating these integrals is that of gaussian quadratures which is described next.

### 5.4 Gaussian Quadrature Methods

For simplicity

$$\Xi \left(n_{1},n_{2},n_{3},n_{4}\right)=\int_{-\infty }^{\infty }d\xi e^{-2\xi^{2}}H_{n_{1}}\left(\xi \right)H_{n_{2}}\left(\xi \right)H_{n_{3}}\left(\xi \right)H_{n_{4}}\left(\xi \right),$$ (87)

may be rewritten, with the transformation $\upsilon =\sqrt{2}\xi$, as

$$\Xi \left(n_{1},n_{2},n_{3},n_{4}\right)=\frac{1}{\sqrt{2}}\int_{-\infty }^{\infty }d\upsilon \;e^{-2\upsilon^{2}}H_{n_{1}}\left(\frac{\upsilon }{\sqrt{2}}\right)H_{n_{2}}\left(\frac{\upsilon }{\sqrt{2}}\right)\times H_{n_{3}}\left(\frac{\upsilon }{\sqrt{2}}\right)H_{n_{4}}\left(\frac{\upsilon }{\sqrt{2}}\right).$$ (88)

Using a gaussian quadrature integration technique [29] reduces this integral to a finite sum

$$\Xi \left(n_{1},n_{2},n_{3},n_{4}\right)=\frac{1}{\sqrt{2}}\sum_{i=0}^{N_{\mathrm{Quad}}^{\left(\xi \right)}}w_{i}^{\left(h\right)}H_{n_{1}}\left(\frac{g_{i}^{\left(h\right)}}{\sqrt{2}}\right)H_{n_{2}}\left(\frac{g_{i}^{\left(h\right)}}{\sqrt{2}}\right)\times H_{n_{3}}\left(\frac{g_{i}^{\left(h\right)}}{\sqrt{2}}\right)H_{n_{4}}\left(\frac{g_{i}^{\left(h\right)}}{\sqrt{2}}\right),$$ (89)

Where $g_{i}^{\left(h\right)}$ is the *i*th Gauss-Hermite quadrature point, and $w_{i}^{\left(h\right)}$ is the corresponding weight. This sum is exactly equal to the integral provided $N_{\mathrm{Quad}}^{\left(\xi \right)}>2$ max(*n_z_*) [29].

Applying the same technique to

$$U\left(\lambda_{1},\lambda_{2},\lambda_{3},\lambda_{4}\right)=\int_{0}^{\infty }d\zeta e^{-2\zeta }\zeta^{\frac{1}{2}\left(m_{1}+m_{2}+m_{3}+m_{4}\right)}\times L_{n_{1}}^{\left(m_{1}\right)}\left(\zeta \right)L_{n_{2}}^{\left(m_{2}\right)}\left(\zeta \right)L_{n_{3}}^{\left(m_{3}\right)}\left(\zeta \right)L_{n_{4}}^{\left(m_{4}\right)}\left(\zeta \right)$$ (90)

with transformation *u* = 2*ζ*, gives the sum

$$\begin{array}{l} U\left(\lambda_{1},\lambda_{2},\lambda_{3},\lambda_{4}\right)=\frac{1}{2\frac{1}{2}\left(m_{1}+m_{2}+m_{3}+m_{4}+3\right)}\\ \times \sum_{i=0}^{N_{\mathrm{Quad}}^{\left(\zeta \right)}}w_{i}^{\left(l\right)}{\left(g_{i}^{\left(l\right)}\right)}^{\frac{1}{2}\left(m_{1}+m_{2}+m_{3}+m_{4}\right)}L_{n_{1}}^{\left(m_{1}\right)}\left(\frac{g_{i}^{\left(l\right)}}{2}\right)\\ \times L_{n_{2}}^{\left(m_{2}\right)}\left(\frac{g_{i}^{\left(l\right)}}{2}\right)L_{n_{3}}^{\left(m_{3}\right)}\left(\frac{g_{i}^{\left(l\right)}}{2}\right)L_{n_{4}}^{\left(m_{4}\right)}\left(\frac{g_{i}^{\left(l\right)}}{2}\right), \end{array}$$ (91)

where $g_{i}^{\left(l\right)}$ is the ith Gauss-Laguerre quadrature point, and $w_{i}^{\left(l\right)}$ is the corresponding weight. This sum is exact provided $N_{\mathrm{Quad}}^{\left(\zeta \right)}>2\max\left(n_{\rho }\right)+\max\left(m\right)$ [29].

This means that the choice of basis-set functions fixes the number of quadrature points needed, e.g., for initial calculations of a cylindrically symmetric ground state condensate, we may use *n_ρ_* = {0, …, 6}, *m* = 0, and *n_z_* = {0, …, 6}, fixing $N_{\mathrm{Quad}}^{\left(\xi \right)}=13$, and $N_{\mathrm{Quad}}^{\left(\zeta \right)}=13$.
